# Identifying Individuals for Integrated Multidisciplinary Care: Lessons from Finland

**DOI:** 10.5334/ijic.6000

**Published:** 2022-08-12

**Authors:** Juha Koivisto, Hanna Tiirinki, Eeva Liukko

**Affiliations:** 1 Finnish Institute for Health and Welfare, FI

**Keywords:** integrated care, multidisciplinary, health and social care, services

## Abstract

**Introduction:**

This paper analyses and discusses the models and tools in the Finnish health and social care system to identify the individuals who might benefit from integrated multidisciplinary care.

**Description:**

The analysis and discussion of the paper is based on a study which mapped and studied the models and tools in practice or under development for identification in the Finnish health and social care organizations. The study used electric survey and structured interviews as research methods.

**Discussion:**

There are several different established models of identification in Finland and the experiences of using them are mainly positive. However, only every third of health and social care organizations in Finland have defined a common model or tool. The identification practices and criteria vary by region, municipality and/or organization. The identification is in general unsystematic and insufficient in practice that may inhibit the individuals to access the integrated care they might benefit from.

**Conclusions:**

Models and tools are needed for founding and identifying individuals who are outside the service system, those whose client-ship has just begun, and those who already access services. The identification of individuals for integrated multidisciplinary care and the assimilation and understanding of different identification models and tools requires the development of basic and further competence in the different fields of health and social care. Multidisciplinary collaboration requires shared concepts and positive attitudes on the development of integrated professional environments, identification models and services. It is therefore also a question of shared working culture.

## Introduction

This paper analyses and discusses the models and tools in the Finnish health and social care system to find and identify the individuals who might benefit from the integrated multidisciplinary care.

Several studies have concluded that about ten percent of the users of the health and social care services use plenty of services and produce about 80 percent of the costs of care. The high costs of care have been observed to be connected to several simultaneous chronic diseases, mental illnesses, and use of specialized health care [1,2,3,4]. This ten percent of users concerned are typically heavy users of health and social care services, but they do not all use services from different service groups or sectors. This paper especially focuses on the multidisciplinary use of health and social care services where a client uses services from the different services groups, such as primary health care, social services, and mental health services.

The reasons for the use of several different health and social services can be manifold. Unemployment, disability pension, substance abuse problems, self-reported declined state of health, chronic diseases and dissatisfaction on the life situation have been observed to generate multidisciplinary service use [5,6]. In the profilations of health and social care service use the clients who use multidisciplinary services have been found to be divided for example into the loaded families with children, to the parents who need special aid and support, and to the substance abuse clients [7]. These clients typically have several health problems and/or social challenges and their general situation in life is complex. Use of the health and social care services of these clients may be uncontrolled and they may use services which do not meet their individual service needs. Nobody is coordinating or guiding their service use or their individual service paths.

At best, the individuals with risk to have several health and/or social problems are found and identified in an early phase when they can be offered early support and help. Then it might be possible to avoid human suffering and uncontrolled use of several different services.

In Finland nearly 200 municipalities and federations of municipalities have organized most primary care and social services and the 21 hospital districts have organized specialized health care during several decades. The Finnish system is usually considered to be effective, efficient, and relatively integrated compared to the case in most other countries. However, at the national level, the system is often evaluated less favorably, and several issues in terms of access to and quality and efficiency of the services have been identified. As the Finnish health and social care system is highly decentralized, with strongly devolved decision-making powers and weak central government steering mechanisms, the provision of health and social care in Finland has become fragmented. Consequently, the national health and social care system has not been able to adequately meet the changing needs of the population, leading to poor and inequitable access to services. In addition, the multidisciplinary use of health and social care services is poorly identified and observed in the fragmented service system [8].

Partly for these reasons the responsibility for organizing the health and social care services will be transferred from the municipalities and the hospital districts to the new 22 wellbeing services counties from the beginning of 2023. The new law which governs the organizing of the health and social care services obliges the new counties to identify the individuals who might benefit from multidisciplinary care, but it does not define any spesific guidelines or criteria how this should be done. Nor do the health care and social welfare acts which oblige to assess the service needs of clients.

The Government of Finland funded the study to which this paper is based on for analyzing the current state of identifying individuals for integrated multidisciplinary care in the Finnish health and social care system and for developing the identification in the new counties.

## Background review, concepts and approach

A recent literature review [9] mapped models and tools for identifying individuals for integrated multidisciplinary care. The review included studies from several countries (excluding Finland) and 64 research publications were analysed. The mapped studies focused above all on the amount of service use, on the frequent use of a specific service and on the costs of service use and principally within health care. The aim of these studies was typically to develop models and tools for anticipating heavy or frequent use of services. The models used a variety of data to predict or identify service needs and demanding health or social situations, such as client and patient databases, questionnaires, self-assessment tools and cost-based forecasts [9].

There were much less studies on the multidisciplinary use of services and on the models and tools for identifying individuals for integrated multidisciplinary care. From our perspective, the models and tools found by the review were to some extent deficient and uninteresting. The multidisciplinary integration of services is at the heart of the health and social care reform being implemented in Finland [10,11]. Therefore, a better understanding of the models and tools for identifying individuals to integrated multidisciplinary care is essential for the development of the Finnish health and social care system.

A client who uses plenty of services quantitatively is typically called a heavy user or frequent user of services. This kind of use is not necessarily multidisciplinary use of services. A client might use for example a lot of services in primary health care. However, there is no absolute definition for what kind of use exactly is heavy or frequent use. In the health care literature, there are several definitions of heavy or frequent use combined to primary health care. According to them a heavy user visits a general practitioner 2–24 times in a year [12,13,14]. In social services it may be more common to talk about the length of service use and long-standing clients than heavy use of services.

Neither is there an absolute definition for multidisciplinary use of services. In this paper the use of services is considered multidisciplinary when a client uses services within more than one service group of health and social care, for example within primary health care and social services. However, with respect to both definitions above, they are always based on classifications, groupings or segmentations of services which change over time, and which vary between organizations, regions, and countries.

Identification of an individual for integrated care is the first step in the service process of a client. In an identification practice the health and social care professionals attempt to find and identify the individuals, who have a challenging situation in life with several health and/or social problems. This kind of identification is always based on some kinds of explicit or implicit criteria, values, principles, presuppositions, laws, and information that is taken for granted. The frame for identification and its criteria and content change over time and space. In addition, an individual whose life situation is defined as challenging does not mean that an individual has multidisciplinary service needs. Service needs are not something that can be observed fully objectively, they are rather something that are negotiated and defined in the interaction between the client and health and social care professionals. After we have chosen the criterions for identification, the individuals who fit to them can possibly be identified.

The practice of identification can be studied with the help of practice research approaches which study practices as *performative* [15,16,17,18,19]. According to them the practices of identification have double character. On the one hand they *identify* individuals for integrated care within the frame and criterions used; on the other hand, they *perform* and *define* the individuals. The performativity of identification means that in the identification practice an individual and his/her service needs are provisionally defined, prioritized, and enacted depending partly on the frame in use. In addition, the practice classifies individuals at least to two different segments. When the frame and its criterions are somewhat different, the individuals might be defined differently, or totally different individuals might be defined for integrated care. Undoubtedly the individuals who are identified and guided to integrated multidisciplinary care more than often benefit from the different services, but this is not the point in the argument. The point is solely to characterize the performative nature of this kind of practices – they are not fully objective endeavors which identify and find individuals for integrated care, but rather contingent, selective, relational, and performative in their nature.

## Mapping the models and tools for identification

The analysis and discussion of this paper is based on a study where we mapped and studied during the spring 2019 the models and tools in practice or under development in the Finnish health and social care system to identify the potential individuals for integrated multidisciplinary care. In the study an electric survey was sent to each primary health care centre and social services centre in Finland, to the Social Insurance Institution of Finland (Kela), to some health/social care non-profit organizations and directly to some networks and professionals. The survey inquired the most typical ways of the organizations to identify individuals for integrated multidisciplinary care, asked to describe the models and tools in use or under development, and mapped where and by which professionals they are used, experiences of their use, and which kind of problems and challenges there are in working with them. Over four hundred organizations or units of health and social care in Finland responded to the survey (n = 441). In addition, some structured interviews were made based on the survey results. The data was analyzed statistically and by using qualitative content analysis.

## Identification in finland

A recent study [20] on the multidisciplinary service use in Finland (population 5, 5 million) found that a small and heterogeneous group of clients use multidisciplinary services. The study divided the health and social care services into seven groups. A bit more than 20 percent of the studied population (n = 1 048 180) did not use health and social care services at all during 2017. In all, 38 percent used only the services of one service group, 29 percent used the services of two service groups, seven percent of three services groups and 2, 5 percent of four services groups. A total of 3800 persons from the study population had used services from more than four service groups. However, the service groups defined in the study were quite extensive, such as primary health care, where in each there can be working different professionals and within each service group a client might use several different services.

### Identification models are useful but deficient

The models analyzed in our study for identifying individuals for integrated multisectoral care most commonly target the elderly, patients with diabetes, cardiovascular or other chronic diseases, and people with substance abuse or mental health problems. The models were based on the client or patient interaction, questionnaires, population surveys, client profiling, self-assessments, and data mining models.

There are limitations to the models that hamper the identification of complex life situations. The models are often developed for either social or health care settings. Only a few of the tools use combined data from the primary and specialized health care sectors. In addition, some models are only suitable for specific client groups.

Although various identification models are available, only one in three of the social and health care organizations in Finland have an established model or tool in place. Usually, the identification takes place either by chance or based on the findings of skilled professionals. Proactive identification is not common, and utilization of the client and patient information systems is limited. There is scarce research knowledge available on the effects of the existing models. Understanding about the workability of the models and the effects they generate is primarily based on the experiences of their practical application.

### Three different situations of identification

The survey made it visible that individuals or clients are encountered in various surroundings and social situations. Thus, one identification model can never cover everything, and different kinds of models are needed if the clients with complex life situations and with several social and/or health problems are to be found and identified sufficiently. Most importantly, identification should be an integral part of the work process and its individual stages. In particular, three different anticipation and identification situations were distinguished based on the survey results.

The most common way to identify individuals for integrated care in Finland is when an individual enters the health and social care system. At that point, the Finnish Health Care Act and the Finnish Social Welfare Act require an assessment of the client’s service or care needs. The survey responses illustrated that the professionals should have a sufficiently broad understanding of the factors influencing the client’s situation so that individual service needs are not defined too narrowly from the perspective of their professional competence alone.

On the other hand, a client’s need for multiple, integrated services may come up while he/she is already receiving services. While working with the client, the professional may recognize that the client´s service needs are not adequately met. In some organizations, the monitoring data from information systems may alert the professionals about excessive, overlapping, or misdirected use of services. Also, it may become apparent that some clients may underuse services.

Sometimes potential individuals for integrated care are encountered outside the service system (e.g., homeless, undiagnosed mental health problems, or untreated substance abuse problems). Their difficulties may not have been recognized, or they may have been unwilling or incapable to receive services. The survey brought up some ways to contact these individuals and understand their social conditions. Social work and youth work in the streets and communities (e.g., outreaching youth or elderly work), and low-threshold services such as open common shared living rooms or meeting places for citizens offer pathways for encountering individuals who might benefit from integrated services. This kind of outreach social services should be recognized and strengthened, according to the survey responses. Also, the Finnish Social Welfare Act obliges certain authorities to contact the municipal social services if there is concern about someone’s wellbeing. The neighbors, friends, or family members may also contact the social services on behalf of their near ones and make the so-called “statement of concern”.

### Four models to identification

The identification models found in the survey can be grouped into four general, partly overlapping categories. First, several identification models are based on initial interviews or service/care needs assessments required by law and conducted by health and social care professionals. These models are utilized when an individual enters the services or when there are concerns or feelings of inadequacy while the client is already receiving services. Assessments are also reviewed at agreed intervals or when necessary. There might be, for example, an apparent increase in the number of client visits, contacts, or use of social or health emergency services. Interviews and service need assessments are conducted in collaboration and interaction with the client. Especially in social services, they may include a thorough mapping of the client’s life situation when considered necessary.

Sometimes tested tools and methods are used during the interview to get more precise information and help to define service needs (e.g., Alcohol Use Disorder Test AUDIT and Beck Depression Inventory BDI). In Pirkanmaa county, the client and the professional assess together client’s resources and health or welfare risks using the so-called Suuntima tool, where the client-ship is then categorized according to the client’s identified service needs, and the service path is defined. The organization can also accumulate client-based data by Suuntima to segment client-ships and service production in broader service system contexts.

Second, some models utilize multidisciplinary teams or paired teams to identify individuals for integrated multidisciplinary services. The teams provide multidisciplinary support to individual professionals, and they are used as needed. They may be convened, for example, if there is concern about a client or if a client is accessing services more frequently, or in cases where a client has an issue that cannot be resolved by a single appointment or by a certain professional. Alternatively, advice can be sought by a designated contact person or other named parties. The client may also be referred to a specialist in the team according to the individual service needs.

Some models bring different professionals and the client to interact together for identifying his/her service needs and for forming a shared vision (e.g., The ABC Model of Multidisciplinary Co-operation). Some models give support to the professionals on addressing multidimensional subjects (e.g., Let’s Talk About Children Model, LTC). The services may also be organized as multi-professional entities by a local decision. Sometimes cooperation is required by law. For example, the cross-sectoral joint service for the long-term unemployed is a national co-operating model for the employment services, health and social care services, and the Social Insurance Institution of Finland.

Third, some models rely on designated professionals (e.g., coordinators or case managers), who are responsible for coordinating joint action, such as referrals, services, and the work of multidisciplinary teams. These professionals are often tasked with finding out about the client’s situation once someone has observed deficiencies or dysfunction elsewhere in the service system. Coordinators investigate the client’s circumstances and service needs and either convene representatives of other services or instruct staff in recognizing those in possible need of multidisciplinary services. It is also often their responsibility to ensure that the client receives services as agreed. For example, North Karelia County uses so-called a Joint Case Manager Model, where designated professionals work with clients who have had difficulties accessing or using health and social care services. The role of the case manager is to map the client’s life situation, draw up an individual plan for care and services and coordinate the implementation of the services. Case manager also acts as a contact person for the client.

Fourth, some identification models include the use of health and social care information systems. They can provide data based on client behavior such as irreversible appointments, number of appointments, addictive behavior, admissions, or health risks. Once the information system alerts or makes it visible that the predetermined criteria are exceeded, a professional can assess the client’s service needs more closely. In the survey responses, annual visitation thresholds ranged from six to ten visits.

The use of health and social care information systems to identify clients or patients for integrated care was less common than the other means of identification; however, they were utilized in some organizations. For example, the City of Helsinki uses the Health Benefit Analysis Tool, which automatically collates risk factors recorded in the patient information system and analyses whether a patient is at risk of increased service needs. North Karelia County has utilized information systems to develop a method for identifying individuals or families who might benefit from several different health and social care services simultaneously.

### Factors that impair identification

The survey showed that identifying clients for integrated multisectoral care is hampered by the inadequate development of information systems, separated working cultures, lack of knowledge about the work and competencies of other sectors and professionals, and lack or inadequacy of established models.

Most information systems do not automatically generate aggregate data, let alone alert professionals about potential clients who might benefit from multidisciplinary services. Thus, professionals are unable to see whether a client accesses multiple services or, for example, frequently visits in emergency or out-of-hours services. Confidentiality regulations were mentioned as an essential barrier to information-sharing between professionals and collating data from different systems. Some clients are reluctant to give permission to share their information to professionals in another sector or service group.

Also, professionals’ attitudes toward a particular tool or lack of knowledge can contribute to the problem. Established identification models are not used systematically. For example, employees are sometimes unaware that such a tool exists or do not know when and how it should be used. There is a lack of knowledge of all the legislation related to client work or the comprehensive range of services available to a client. The implementation of approaches, such as service needs assessment and multidisciplinary cooperation, was also found to require specific expertise from the professionals. Nevertheless, the survey results indicated that the experiences of using the established models were fairly positive.

## Discussion

In this paper we have discussed and described by which kinds of models and tools the individuals are identified for integrated multidisciplinary care. In Finland the identification practices and criteria seem to vary a lot by region, municipality, and organization [7,21]. Our analysis implies and emphasizes several development activities of identification models for the new wellbeing services counties starting from the beginning of 2023 in Finland. It is important to note that identifying multidisciplinary service needs is also a matter of equal access to services

We argue that there should be a comprehensive and uniform set of identification models and tools in use in a county for identifying the individuals in different kinds of situations: those who are outside the service system, those whose client-ship has just begun, and those who already access services [21]. In Finland, those whose client-ship has just begun are identified most, because the current health and social care legislation requires service or care need assessment. Also, those who are already accessing services are usually identified when there are changes in their life situation or when there are challenges in their use of services, for example frequent emergency calls or repeated visits.

It is more difficult to identify individuals who are outside the service system and who might benefit from integrated care. However, especially they should be guaranteed equal access to the services they might benefit from. The tools could include screening of the risk factors at the population level, outreach social work, and community work [21]. To make this happen more systematic use of individual and demographic data from health and social care information systems is required. Also, development of new identification models and better use of existing ones, such as outreach young or elderly social work, is needed.

It seems clear that the professionals would benefit from the automatic alerts and signals concerning the clients and their service use when attempting to identify individuals for integrated care [21]. Our finding is that they are not sufficiently exploited in Finland. The utilization of digitalization should be studied and developed to support the identification, anticipation, and decision-making. The uniform entering of client data on the information systems should be developed so that in the identification practice the health and social care registries could be utilized more effectively. Because of this, the Finnish health and social care legislation should also be developed to provide chances for the multidisciplinary collaboration in practice.

The findings of our analysis imply that it is urgent to enhance and systematize the multidisciplinary collaboration, information-sharing, and identification practices in the new counties. Currently, there is often too much responsibility on single professionals to identify the clients who would benefit from integrated services and to refer them to appropriate services. Often identification is based on personal competence rather than a systematic approach. Timely identification of multidisciplinary service needs is also likely to reduce the costs of services when the uncoordinated use of services can be avoided [1,2,7,20].

The identification of individuals for integrated multidisciplinary care is too often seen as a separate phase of work, although it should be an integral part of the whole work process [7,21]. Also, client-centeredness and interactive cooperation with clients should be strengthened. In general, it would be advisable to have a so-called named person for clients with multidisciplinary service needs who supports and guides the individual through the integrated service path. The responsibility for accessing services should not remain with the individual when his/her personal resources may be otherwise weak.

Based on the literature review of Kivipelto et al, [9]. we argue that despite the challenges tackled in this paper Finland is a forerunner in the identification and/or anticipation of individuals for integrated multidisciplinary care, while in many countries the focus is on the frequent use of a specific health service. Thus, the suggestions of this paper for developing the identification practice are informative for the other countries as well.

## Lessons learnt

- In the current situation models for identifying individuals for integrated multidisciplinary care are not implemented and used systematically in the health and social care system in Finland.
- There should be a comprehensive and uniform set of identification models and tools in use in each county to identify individuals in different kinds of situations.
- The development of identification models and tools should be part of a broader strengthening of the client-centeredness of health and social care services.
- The utilization of digitalization and information systems for the identification and anticipation of service needs should be studied and developed extensively to support the decision-making.
- Collaboration between different professionals is a common ground and precondition for the identification practice, the post-identification process, and the coordination of an integrated service path.

## Conclusions

Based on our study concerning Finland, some general conclusions can be drawn. The identification of individuals for integrated multidisciplinary care and the assimilation and understanding of different identification models and tools requires the development of basic and further competences in the different fields of health and social care. Multidisciplinary collaboration requires shared concepts and positive attitudes to the development of integrated professional environments, identification models and services. It is therefore also a question of shared working culture. The adoption of new models and work roles, such as case managers and shared expertise, requires from the professionals respect to each other, professional perspectives, extensive competence, and knowledge of the service system. Multidisciplinary dialogue between health and social care professionals is required to strengthen the understanding of those who might benefit from integrated care.

A potential limitation of our study was that only an electric survey and some additional interviews were conducted. However, the number of organizations which responded to the survey was comprehensive. The survey results gave a general picture of the identification models and tools in use in Finland. The additional interviews increased our understanding of the use and applicability of the models and tools in practice. However, further research is needed. A case study of identification in action would give a deeper and richer understanding of how the individuals are identified and performed by the models or tools, how they are classified, who are left unrecognized, and what kinds of consequences the identification practices generate.

## Additional Files

The additional files for this article can be found as follows:

10.5334/ijic.6000.s1 Supplementary file 1. Three situations of identification.

10.5334/ijic.6000.s2 Supplementary file 2. The process of a client in the integrated multidisciplinary care.
