# Supplementary figures and images for: Identifying Individuals for Integrated Multidisciplinary Care: Lessons from Finland
Source: Int J Integr Care. 2022 Aug 12;22(3):8. doi: 10.5334/ijic.6000 (PMC9374024; doi:10.5334/ijic.6000)

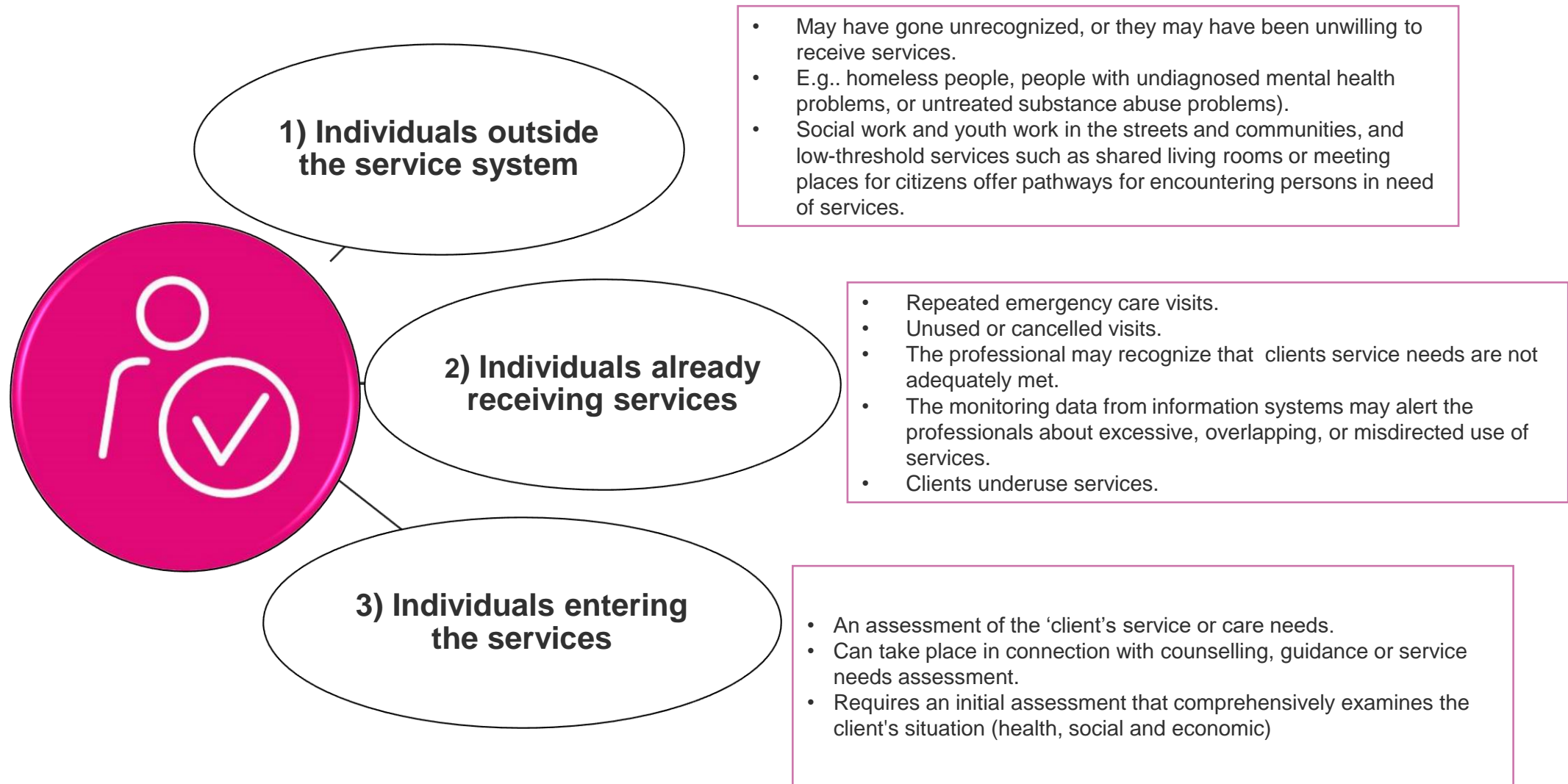

Supplement: Supplementary file 1. — Three situations of identification. [file ijic-22-3-6000-s1.pdf]
