# Supplementary material for: Identifying Individuals for Integrated Multidisciplinary Care: Lessons from Finland
Source: Int J Integr Care. 2022 Aug 12;22(3):8. doi: 10.5334/ijic.6000 (PMC9374024; doi:10.5334/ijic.6000)
Supplement: Supplementary file 2. — The process of a client in the integrated multidisciplinary care. [file ijic-22-3-6000-s2.pdf]

INDIVIDUALS OUTSIDE THE SERVICE SYSTEM

INDIVIDUALS ENTERING THE SERVICES

INDIVIDUALS ALREADY RECEIVING SERVICES

Utilization of identification models and tools

Anticipating service needs	Digital tools	Interactive work	Information systems	Contacts and notifications
Risk assessment<br>Exploratory work<br>Population-level information<br>Aggregate-level identification and anticipation of risk groups	Digital tools (e.g. symptom assessments)<br>Electronic health checks	Initial interview<br>Service needs assessment<br>Patient segmentation<br>Low threshold services<br>Counselling and guidance<br>Health checks e.g. for the unemployed	Measurements<br>Alarm limits, triggers<br>Changes in social and health care service use	Contacts by family members or near ones,<br>Child welfare notifications<br>Notifications of the need for social welfare services

Coordination of services

INDIVIDUAL SERVICE PATH

No need for services
Monitoring if needed

Early support and preventive services
If necessary, designation of a responsible worker/professional

Multidisciplinary service need assessment together with the client
Designation of the responsible worker/professional

Monitoring and evaluation

CREATION OF INDIVIDUAL AND MULTIDISIPLINARY PLAN BASED ON SERVICE NEEDS, UTILIZING SERVICE PACKAGES AND CHAINS → IMPLEMENTATION OF INDIVIDUAL SERVICE PATH PLAN

Impact of multidisciplinary services to the individual well-being, health and quality of life
